# Risperidone induced neutropenia in a 75-year-old man

**DOI:** 10.1192/j.eurpsy.2022.1869

**Published:** 2022-09-01

**Authors:** D. Nistor, L. Horosan, A. Giurgiuca

**Affiliations:** 1 “Prof. Dr. Al. Obregia” Psychiatry Hospital, General Psychiatry, Bucharest, Romania; 2 University of Medicine and Pharmacy Carol Davila, Neuroscience 6, Bucharest, Romania

**Keywords:** Antipsychotics, neutropenia, risperidone, Side effects

## Abstract

**Introduction:**

We discuss the case of a 75-year-old man with no psychiatric history, presenting with complex auditory hallucinations, both commentary and imperative, delusions of persecution and prejudice, severe anxiety, modified behaviour, and altered sleep patterns.

**Objectives:**

The patient was started on oral risperidone, with favourable evolution of symptoms after reaching a daily dose of 3 mg/day. After three weeks of treatment, the laboratory results showed a low number of neutrophils. Interdisciplinary approach and examinations which included both clinical and paraclinical evaluation concluded that another cause of neutropenia was highly unlikely.

**Methods:**

The patient was switched to olanzapine, with gradually increasing doses up to 10 mg/day. A significant improvement of the neutrophils’ level was noticed, with a return to normal parameters after a few days. Nevertheless, the clinical course was unfavourable, with reoccurrence of auditory hallucinations and delusions in two weeks’ time. Decision to rechallenge was made, with careful monitoring of the blood test results, particularly neutrophil levels. Risperidone was started at low doses of 0.5 mg/day and gradually increased up to 2 mg/day.

**Results:**

Seven days after risperidone reinitiation laboratory tests showed normal absolute neutrophil count. However, another week later, neutrophils fell again out of the normal range.

**Conclusions:**

The patient was discharged with haloperidol, with adequate control of symptoms and no adverse reactions.

**Disclosure:**

No significant relationships.

